# Effervescent Salts

**Published:** 1890-05

**Authors:** G. W. Pickerill


### Selections and Abstracts.

### EFFERVESCENT SALTS.

There are many late achievements in pharmacy, making the life of the physician very much more pleasant not only to himself, but also to his patients. In this line the "Granular Effervescent Salts" take high rank for "beauty," agreeableness and value as therapeutic agents. Being attractive to the eye, generally pleasant to taste, and agreeable to the most delicate stomach, they have a strong backing for commendation.

I wish especially to call attention to a few of these elegant preparations, those which have been constant fixtures in my office out-fit for daily use for the past four or five years.

Effervescent Bromo Soda. (W. R. Warner & Co.) This is a combination of Caffeine gr. i. and Bromide Sodium grs. xxx. After its use personally for several years, and prescribing it in a large number of cases, I must be pardoned if I speak enthusiastic of it in nervous headache. This difficulty being so often met with a prompt, pleasant and effectual remedy is a boon indeed. This the physician has in Bromo Soda. A nervous headache, resulting from over-work, study, worry, debility, etc., from one to three doses of Bromo Soda will in a very short time put new life and vigor in the sufferer.

From personal experience I can speak of this agent in the most positive terms. And that is, its almost magic effects after it has been necessary to use an opiate for some time, until that peculiar disagreeable sensation, so often felt in the brain, is produced. A dose of Bromo Soda drives this sensation from the brain almost as rapidly as the sun will a "fog" from dark places. The sensation to the patient reminds him of a mist disappearing at the approach of sun light. The head is left as "clear as a bell" in a few minutes.

A teaspoonful in half a glass of sweetened water, drank at once, is a very grateful, sparkling drink.

Granular Effervescent Citrate of Magnesia is another preparation for superior worth. Far superior to the usual liquid form.

"Crab Orchard Salt," an exact analysis of the Crab Orchard Spring, producing the effect of that valuable agent.

Messrs. W. R. Warner & Co., have presented to the profession a long list of "Effervescent Salts," many of them of superior value as therapeutic preparations.—*Medical Free Press.*

DR. G. W. PICKERILL.

